# Identification and Characterisation of the Early Differentiating Cells in Neural Differentiation of Human Embryonic Stem Cells

**DOI:** 10.1371/journal.pone.0037129

**Published:** 2012-05-15

**Authors:** Parinya Noisa, Thamil Selvee Ramasamy, Fiona R. Lamont, Jason S. L. Yu, Michael J. Sheldon, Alison Russell, Xin Jin, Wei Cui

**Affiliations:** Department of Surgery and Cancer, Faculty of Medicine, Institute of Reproductive and Developmental Biology, Imperial College London, London, United Kingdom; Genome Institute of Singapore, Singapore

## Abstract

One of the challenges in studying early differentiation of human embryonic stem cells (hESCs) is being able to discriminate the initial differentiated cells from the original pluripotent stem cells and their committed progenies. It remains unclear how a pluripotent stem cell becomes a lineage-specific cell type during early development, and how, or if, pluripotent genes, such as Oct4 and Sox2, play a role in this transition. Here, by studying the dynamic changes in the expression of embryonic surface antigens, we identified the sequential loss of Tra-1-81 and SSEA4 during hESC neural differentiation and isolated a transient Tra-1-81(−)/SSEA4(+) (TR−/S4+) cell population in the early stage of neural differentiation. These cells are distinct from both undifferentiated hESCs and their committed neural progenitor cells (NPCs) in their gene expression profiles and response to extracellular signalling; they co-express both the pluripotent gene Oct4 and the neural marker Pax6. Furthermore, these TR−/S4+ cells are able to produce cells of both neural and non-neural lineages, depending on their environmental cues. Our results demonstrate that expression of the pluripotent factor Oct4 is progressively downregulated and is accompanied by the gradual upregulation of neural genes, whereas the pluripotent factor Sox2 is consistently expressed at high levels, indicating that these pluripotent factors may play different roles in the regulation of neural differentiation. The identification of TR-S4+ cells provides a cell model for further elucidation of the molecular mechanisms underlying hESC neural differentiation.

## Introduction

The developmental processes of many organs and tissues in an embryo originate from the pluripotent cells of the inner cell mass (ICM) in the blastocyst. As development proceeds, these cells gradually acquire specialized traits, becoming committed to specific fates and losing their potential to differentiate into other cell types. For example, the development of the central nervous system is initiated following gastrulation by the induction of the neuroectoderm, a process by which embryonic cells acquire a neural fate to form a single layer of neuroepithelial cells [1]. These cells subsequently give rise to neural stem and progenitor cells, which undergo further differentiation to neurons and glia [2]. This multi-step cell fate determination that occurs during embryonic neurogenesis is delicately orchestrated by many signalling pathways and transcription factors. Although considerable efforts have been focused on ascertaining the emergence of these earliest potential neural cells and the regulatory mechanisms that govern the process of neural induction, they have yet to be fully defined. This is largely due to the lack of adequate tissues from the early developmental stages.

Human embryonic stem cells (hESCs) derived from the ICM of blastocysts are capable of self-renewal in culture indefinitely and meanwhile retain the developmental pluripotency of the embryonic founder cells, having the potential to differentiate into all the cells and tissues in a human body [3]. Therefore, they provide not only a potential source of specialized cells for regenerative therapies but also a valuable *in vitro* model to study early human development, particularly as the direct study of early human embryo development is severely hampered by the inability to obtain adequate amounts of tissues at all developmental stages. Although differentiation of ESCs may not fully recapitulate the development of the embryo, increasing evidence demonstrates that their lineage-specific differentiation nonetheless reflects the developmental progression of that cell type *in vivo* [4]–[7]. Therefore, the use of hESCs to investigate early human embryo development may provide valuable insights into early developmental processes, including neural induction.

The Oct4 transcription factor plays an essential role in the maintenance of pluripotency and self-renewal of ESCs [8], [9] and is also a critical reprogramming factor [10]. In mouse, Oct4 is initially expressed in all the blastomeres of the morula, with its expression becoming successively restricted to the ICM of the blastocyst. After gastrulation, the expression of Oct4 is concentrated in the primitive ectoderm and persists through E7.5 in unsegmented areas, but is downregulated as development continues. By E9.5, its expression is limited to primordial germ cells [11]. In human, Oct4 expression remained at stage 9 post-implantation embryos [12]. In the absence of Oct4, embryos are unable to form pluripotent ICM and fail to produce any other lineages, except for extraembryonic trophoblasts [13]. Correspondingly, forced downregulation of Oct4 in mouse and human ESCs results in their differentiation into extraembryonic lineages [8], [14], [15]. Taken together, this implies that Oct4 plays a significant role in embryogenesis and early lineage differentiation.

We have previously shown that hESCs can be efficiently differentiated to neural progenitors by the inhibition of BMP [16]. During the initial differentiation, expression of Oct4 remains detectable for at least one week until the formation of neuroepithelial cells after week 2. Little is known about these early differentiating cells and it is not clear whether this initial Oct4 expressing population differ from undifferentiated hESCs. In this study, we carefully identified and isolated this initial differentiating cell population and demonstrated that these cells are distinct from undifferentiated hESCs and committed neural progenitor cells (NPCs), exhibiting intermediate features between the two. The identification of these early neural differentiating cells will provide a valuable cell source which can be used to elucidate the molecular mechanisms that regulate neuroectoderm development.

## Results

### Identification of a transient Tra-1-81(−)/SSEA4(+) cell population at early stage neural differentiation of hESCs

Similar to the cells in the ICM, undifferentiated hESCs express embryonic cell specific surface antigens, including stage-specific embryonic antigens (SSEA) 3 and 4, Tra-1-60 and Tra-1-81 [17], which were first identified in human embryonic carcinomas. However, unlike mESCs, they do not express SSEA1. Although the functional significance of these antigens is as yet unclear, they are routinely used as markers for hESCs. Therefore, we anticipated that studying the dynamic changes of these cell surface markers in neural differentiation could potentially enable us to capture and isolate the early differentiating populations which could be used for further analysis.

**Figure 1:**
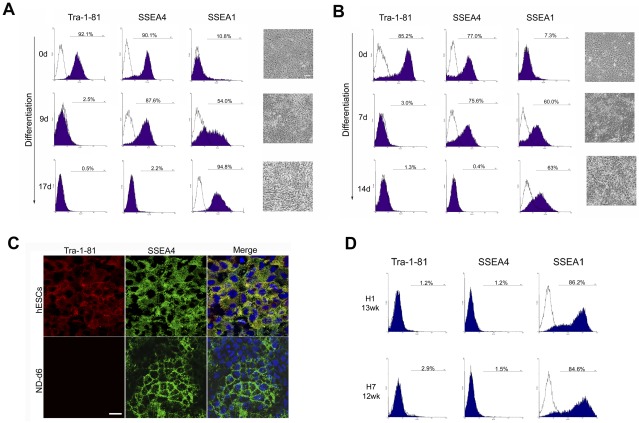
Sequential loss of Tra-1-81 and SSEA4 expression during neural differentiation of hESCs. (A) & (B) Expression of Tra-1-81, SSEA4 and SSEA1 during neural differentiation of H1 (A) & H7 (B) hESCs respectively on the indicated days of differentiation. Phase-contrast images are shown. Scale bar = 50 µm. (C) Immunostaining of Tra-1-81 and SSEA4 in H7 hESCs and at day 6 of their neural differentiation. Scale bar = 20 µm. (D) Expression of Tra-1-81, SSEA4 and SSEA1 in hESC-derived neural progenitor cells. Days of the differentiation are indicated.

We focused our study on Tra-1-81, SSEA4 and SSEA1, given that SSEA3 expression is not necessarily required for a pluripotent state [18], and the high similarity between Tra-1-60 and Tra-1-81 [19]. Our results showed that, in self-renewal culture conditions, H1 hESCs express high levels of SSEA4 and TRA-1-81, but not SSEA1 (Figure 1A, top). After differentiation to neural progenitors, expression of Tra-1-81 and SSEA4 were lost while expression of SSEA1 was increased (Figures 1A, bottom). However, it is noteworthy that the loss of Tra-1-81 preceded that of SSEA4. After 9 days in neural differentiation medium, Tra-1-81 expression was almost completely lost, whereas the majority of cells still expressed SSEA4 (Figure 1A, middle). The expression of SSEA4 persisted for several days until 2 weeks into the differentiation at which point the neuroepithelial cells started to emerge and SSEA1 expression was positive. A similar pattern was also observed in H7 hESCs, although Tra-1-81 expression was lost earlier than that in the H1 cells (7 days *vs.* 9 days) (Figure 1B). The existence of this Tra-1-81(−)/SSEA4(+) population of cells was also confirmed by immunostaining (Figure 1C). Further neural differentiation from this stage lead to the efficient generation of NPCs, which retained the expression pattern of Tra-1-81(−)/SSEA4(−)/SSEA1(+) and could be maintained for an extended time in culture when supplemented with bFGF/EGF (Figure 1D). The neural differentiation experiments were repeated several times in both H1 and H7 hESC lines and the sequential loss of the Tra-1-81 and SSEA4 antigens was reproducible, although the timing of the disappearance of each antigen varied slightly between experiments, depending on the initial seeding density.

To eliminate the possibility that the sequential loss of Tra-1-81 and SSEA4 is a culture-dependent phenomenon, hESCs were also differentiated using the double SMADs inhibition protocol [20], [21]. Differentiation with the dual SMAD inhibitors exhibited the same sequential loss of Tra-1-81 and SSEA4 in both H1 and H7 cells (Figure S1A). Furthermore, a previous report using stromal-feeder based neural differentiation protocol observed the same effect [19]. We therefore propose that this initial Tra-1-81(−) and SSEA4(+) population represent cells of early neural differentiation and consequently designated them TR−/S4+ cells.

### TR−/S4+ cells exhibit an intermediate gene expression pattern between hESCs and neural progenitors

**Figure 2:**
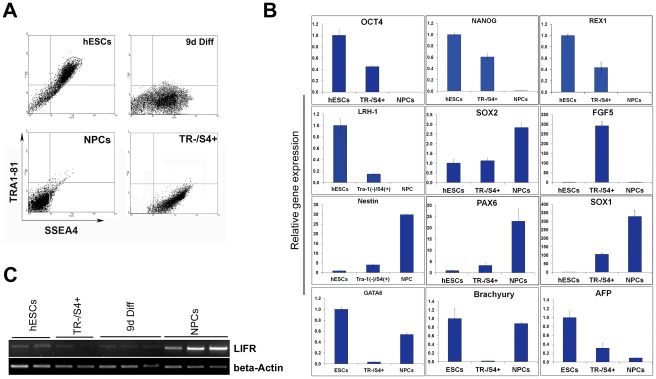
Gene expression profile in undifferentiated hESCs, TR−/S4+ cells and neural progenitor cells (NPCs). (A) Flow cytometry analysis of cells co-stained with Tra-1-81 and SSEA4 antibodies in the three stages of neural differentiation and in purified TR−/S4+ cells. (B) qRT-PCR analysis of marker gene expressions in TR−/S4+ cells, hESCs and NPCs. Standard deviations were calculated from at least three independent experiments. (C) RT-PCR analysis of LIFR in undifferentiated H1 hESCs, sorted TR−/S4+ cells, unsorted day 9 differentiated cells (9 d diff) and NPCs. Each lane represents an independent experiment.

Different cell populations can be distinguished by morphological and developmental criteria, as well as by the temporal and spatial expression of marker genes. In order to characterize the transient TR−/S4+ cells, we isolated them by FACS or by magnetic-activated cell purification (Figure 2A). The Tra-1-81(−) and SSEA4(+) identity of the purified cells was then confirmed by flow cytometry analysis. Gene expression was analysed by quantitative RT-PCR (qRT-PCR) and compared to that of undifferentiated TR+/S4+ hESCs and their TR−/S4− NPCs (Figure 2B). We found that the expression of pluripotent marker genes, *Oct4*, *Nanog* and *Rex1*, was clearly downregulated in the TR−/S4+ cells compared to hESCs but remained higher than that of NPCs. Conversely, the expression of neural progenitor marker genes, *Pax6*, *nestin* and *Sox1*, was upregulated in the TR−/S4+ cells but was considerably lower than in NPCs. However, Sox2, another known pluripotent marker, was consistently expressed in all three cell types, with its highest expression level in the NPCs, implicating a critical role in both pluripotent and neural progenitor cells. In addition, the TR−/S4+ cells also expressed the early differentiation marker, *FGF5*, which was undetected in both hESCs and NPCs (Figure 2B). A similar gene expression pattern was also found in TR−/S4+ cells from neural differentiation with dual SMAD inhibitors. It was also found by RT-PCR that, compared to the undifferentiated hESCs and their NPCS derivatives, both the isolated TR−/S4+ cells and the unsorted day 9 differentiated H1 cells expressed much lower levels of leukaemia inhibitory factor receptor (LIFR) (Figure 2C).

**Figure 3:**
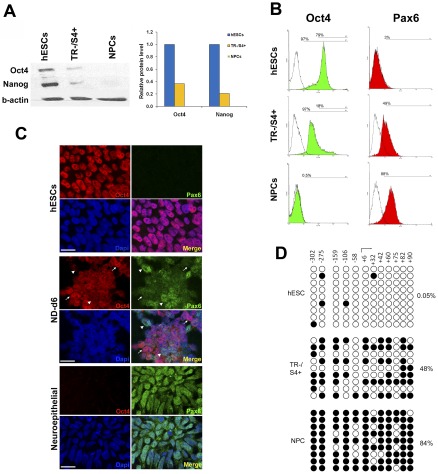
Oct4 and Pax6 expression in hESCs, TR−/S4+ cells and NPCs. (A) Western blotting image and quantitative histogram showing Oct4 and Nanog protein expression in the three cell types. (B) Flow cytometry analysis of Oct4 and Pax6 expression in the three cell types. Percentages of total Oct4 positive and high Oct4 expressing cells are indicated. (C) Immunostaining of Oct4 and Pax6 proteins in H7 hESCs, day 6 neural differentiation (ND-d6) and rosette-forming neuroepithelial cells. Scale bar = 20 µm. Arrows indicate cells with high Oct4 and low Pax6 expression; arrowheads indicate cells with high Pax6 and low Oct4. (D) Bisulphite DNA sequencing of the *Oct4* promoter region in hESCs, TR−/S4+ cells and NPCs. The transcription starting site and the corresponding location of CpG are indicated. Open and closed circles indicate unmethylated and methylated CpG, respectively.

Western blotting was performed to confirm the expression of Oct4 and Nanog proteins (Figure 3A). While undifferentiated hESCs exhibited high levels of Oct4 and Nanog proteins, they were undetectable in NPCs. However, in the TR−/S4+ cells, Oct4 and Nanog protein levels were lower than in hESCs but higher than in the NPCs. Since both RT-PCR and Western blotting methods quantify average levels of gene expression in a whole population, one cannot exclude the possibility that this TR−/S4+ population contains two groups of cells: one similar to the undifferentiated hESCs (high Oct4 expression but low/no expression of neural markers), and the other similar to the NPCs (high levels of neural markers but no Oct4 expression). Therefore, to confirm that the expression of Oct4 and Pax6 in the TR−/S4+ cells was not due to the co-existence of these two different populations, immunostaining and flow cytometry analysis were carried out. The results showed that Oct4 antibody staining was positive in 97% of hESCs, with almost 80% cells strongly positive (mean value = 156.5). Similarly, 97% of TR−/S4+ cells were also stained positive for Oct4, but the majority of cells exhibited a lower level of Oct4 (mean value = 54.49, Figure 3B, left). No clear Oct4 staining was visible in NPCs (mean value = 2.9). In addition, TR−/S4+ cells showed a clear upregulation of Pax6 signals (mean value = 12.4) compared to hESCs (mean value = 3.14), which was not as strong as in the NPCs (mean value = 21.1, Figure 3B, right). Co-staining of Oct4 and Pax6 *in situ* confirmed the flow cytometry analysis and clearly demonstrates the co-existence of Oct4 and Pax6 proteins in the same cell during early neural differentiation, which are notably expressed in a negatively correlated manner (Figure 3C).

To explore whether the various levels of Oct4 expression are regulated at the promoter, we examined the amount of DNA methylation in the *Oct4* promoter region near the transcription start site. A gradual increase in DNA methylation was observed throughout the neural differentiation and was negatively correlated with Oct4 mRNA expression (Figure 3D). In hESCs, very low levels of methylation were detected (0.05%), opposed to the heavy methylation detected in NPCs (84.3%). Interestingly, an intermediate DNA methylation level of 48.1% was observed in the TR−/S4+ cells. Taken together, these results demonstrate that the TR−/S4+ cells exhibit a gene expression profile which is distinct from both undifferentiated hESCs and their fully committed neural progenitors, and co-express both neural progenitor and undifferentiated pluripotent markers. In addition, Oct4 expression is progressively downregulated during neural differentiation, which is accompanied by the upregulation of neural markers.

### TR−/S4+ cells generate both neural and non-neural lineage cell types

**Figure 4:**
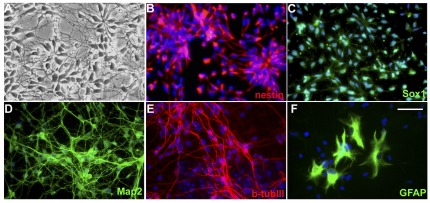
Neural differentiation of TR−/S4+ cells. TR−/S4+ cells were further differentiated for in N2B27 medium supplemented with noggin for 1–2 weeks, then without noggin. (A) Phase-contrast images of further culture of TR−/S4+ in neural differentiation media for 3–4 weeks. (B–F) Immunostaining with the indicated antibodies at different time points: nestin and Sox1 (3–4 weeks), MAP2 and β-tublin III (6 weeks) and GFAP (15 weeks). Scale bar represent 100 µm.

Under neural differentiation conditions [16], further culture of the TR−/S4+ cells lead to the efficient production of NPCs that express high levels of neural progenitor markers: nestin (97%), Sox1 (82%) and Pax6 (88%) (Figure 3 and 4) but lack expression of pluripotent genes (*Oct4* and *Nanog*) or mesoderm and endoderm markers: GATA6, brachyury and α-fetoprotein (Figure 2) [22]. These neural progenitor cells were able to further differentiate into neurons and astrocytes as revealed by the positive staining of MAP2, TUJ1 and GFAP, respectively (Figure 4).

**Figure 5:**
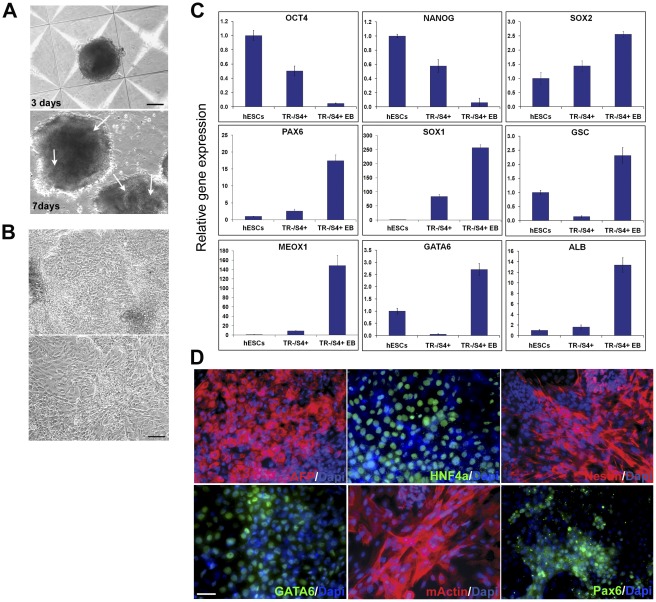
Differentiation of TR−/S4+ cells by cell aggregate formation. (A) Images of cell aggregates at day 3 and 7 of differentiation, respectively. EB-like structures are visible by day 7 (arrows). Scale bar = 100 µm. (B) Images of cell aggregates cultured for a further 7 days after disassociation. Scale bar = 100 µm. (C) qRT-PCR analysis of markers in hESCs, TR−/S4+ and their differentiated progeny (TR−/S4+ EB) two weeks after initiation of differentiation (1 week in suspension and 1 week after dissociation onto adherent dish). (D) Immunostaining with indicated antibodies on differentiated progeny of TR−/S4+ cells 1 week after dissociation onto coverslip. Scale bar = 50 µm.

Since Oct4 expression persists in TR−/S4+ cells, albeit at a lower level, we considered whether these cells were able to generate non-neural lineages in alternative culture conditions. Purified TR−/S4+ cells were differentiated via cell aggregate formation, a method that can initiate spontaneous differentiation and is widely used to examine the differentiation potential of cells *in vitro* [23]. The TR−/S4+ cell aggregates were able to expand in culture and displayed structures similar to embryoid bodies (EBs) after 7 days of differentiation in suspension (Figure 5A). These EB-like aggregates were then dissociated and plated onto adherent culture dishes for further differentiation. After another 7 days, cells with various morphologies were visible (Figure 5B) and were analysed for gene expression by qRT-PCR and immunocytochemistry. Real-time RT-PCR showed upregulation of markers of the three germ layers: neuroectoderm (Pax6, Sox1 and Sox2), mesoderm (goosecoid (GSC) and Meox1) and endoderm (albumin (ALB) and GATA6) (Figure 5C), while pluripotent markers, Oct4 and Nanog were downregulated. Immunocytochemistry further confirmed that the differentiated cells contained progeny from the three germ layers: AFP, HNF4α and GATA6 for endoderm, muscle actin for mesoderm, nestin and Pax6 for neuroectoderm (Figure 5D). These results demonstrate that TR−/S4+ cells are also capable of differentiating into cells of non-neural lineages.

### TR−/S4+ cells cannot be maintained or reverted back to hESCs in self-renewal culture conditions

**Figure 6:**
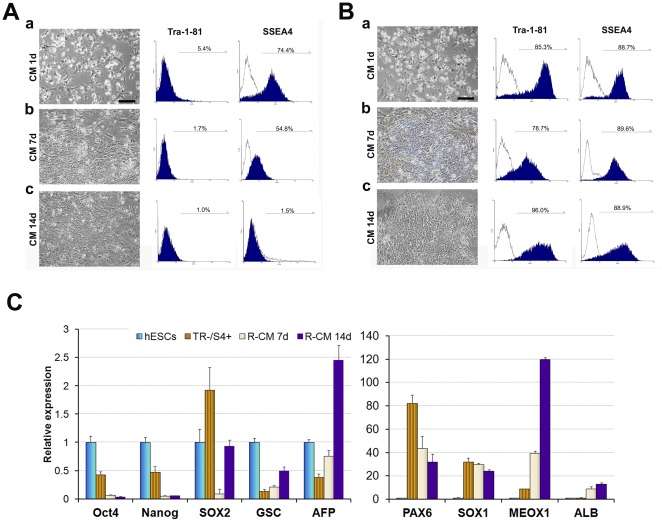
TR−/S4+ cells cultured in MEF-CM supplemented with bFGF. (A) & (B) TR−/S4+ cells (A) and TR+/S4+ hESCs (B) were isolated and cultured in hESC self-renewal conditions for 1 (a), 7 (b) and 14 (c) days. Phase-contrast images and flow cytometry analysis of Tra-1-81 and SSEA4 expression are shown in the left and right, respectively. Scale bar = 100 µm. (C) qRT-PCR analysis of marker genes in TR−/S4+ cells after 0 (TR−/S4+), 7 (R-CM7d) and 14 (R-CM14d) days of culture in MEF-CM hESC culture conditions compared with hESCs.

TR−/S4+ cells express a high level of FGF5, a marker of post-implantation primitive ectoderm of mouse embryos [24]. Given that mESC-derived primitive ectoderm-like cells are capable of reverting back to an ESC state when re-cultured in mESC self-renewal media [25], we examined whether the TR−/S4+ cells can be reverted back to their original hESC state. Purified TR−/S4+ cells were re-plated into matrigel-coated plates and cultured in hESC self-renewal conditions (Figure 6A). To verify that any resulting changes were not a technical artifact, we purified and cultured TR+/S4+ hESCs as controls (Figure 6B). Both cell populations showed similar attachment efficiencies (Figure 6Aa & 6Ba) and colonies were clearly visible 3 days after plating. By day 7, TR−/S4+ colonies exhibited structures and morphologies similar to that of hESCs but the expression of Tra-1-81 and SSEA4 could neither be reactivated nor maintained (Figure 6Ab). In contrast, control hESCs (TR+/S4+) expressed high levels of both these markers (Figure 6Bb). This difference became even more apparent after 14 days of culture (Figure 6Ac & 6Bc). Consistent with the changes in cell surface markers, evident differentiation also started to emerge in the TR−/S4+ cells. RT-PCR analysis confirmed that expression of both Oct4 and Nanog was downregulated in TR−/S4+ cells as the culture proceeded. Mesoderm and endoderm markers were up-regulated while the expression of neural markers, Pax6 and Sox1, was reduced, although the levels remained higher than in hESCs (Figure 6C). These results are consistent with the data from EB cultures, which indicate that the cell fate of TR−/S4+ cells are affected by their culture conditions. In CM cultures, there is limited inhibition of BMP signaling in cells, at least not to the level induced by the addition of noggin [16], which may account for the observed non-neural lineage differentiation. These results further confirm that TR−/S4+ cells are distinct from undifferentiated hESCs and cannot be reverted back to their original hESC state by reintroduction into hESC self-renewal conditions. Furthermore, these cells cannot be maintained as TR−/S4+ cells under these conditions.

### Comparison of TR−/S4+ cells with cells of early embryoid bodies and definitive endoderm differentiation

Since TR−/S4+ cells are able to generate cells in addition to the neural lineage, it raises the question of whether these cells are equivalent to those of early EBs. To answer this question, we compared our global gene expression data of early neural differentiation (N1), which resembled the TR−/S4+ cells (over 85% cells are TR−/S4+) [22], with the published microarray data from 16-day EBs of the same hESC line, H1 [26]. Interestingly, gene ontology analysis revealed that the genes significantly upregulated during EB differentiation have considerable functions during neural differentiation and development (Table 1) and, as a result, shared >60% of the functions in biological processes with those genes upregulated in TR−/S4+ cells. This becomes more evident when the top 15 functions in the biological process of the gene-ontology list are compared between the two cell-populations as over 70% of them appeared in both lists (Table 1). Similarly, the downregulated genes in both cell populations also shared >60% of gene ontology functions. Therefore, global gene expression analysis does provide certain support for the similar phenotype and differentiation observed between TR−/S4+ cells and the early EBs (EBs in suspension).

**Table 1:** Top 15 functions of the genes up- or down- regulated in TR−/S4+ and early EB cells.

	TR−/S4+ upregulated genes *	Benjamini		EB upregulated genes	Benjamini
1	multicellular organismal process (2)	1.40E-22	1	nervous system development	1.60E-14
2	system development (3)	6.30E-12	2	multicellular organismal process	5.40E-13
3	multicellular organismal development (5)	7.90E-12	3	system development	2.30E-12
4	anatomical structure development (4)	1.10E-11	4	anatomical structure development	6.30E-12
5	system process (20)	2.20E-11	5	multicellular organismal development	7.00E-11
6	developmental process (6)	2.70E-10	6	developmental process	1.80E-10
7	cell surface receptor linked signal transduction	4.90E-09	7	central nervous system development	2.30E-08
8	nervous system development (1)	4.70E-08	8	cell differentiation	5.50E-07
9	neurological system process (23)	1.10E-07	9	neurogenesis	5.60E-07
10	G-protein coupled receptor protein signaling pathway	1.80E-07	10	axonogenesis	6.70E-07
11	organ development	2.20E-07	11	cellular developmental process	7.40E-07
12	cellular developmental process (11)	5.40E-06	12	cell morphogenesis involved in neuron differentiation	2.50E-06
13	cell differentiation (8)	5.10E-06	13	generation of neurons	2.50E-06
14	cognition	5.50E-06	14	cell projection morphogenesis	3.30E-06
15	central nervous system development (7)	5.30E-06	15	neuron projection morphogenesis	4.20E-06

	TR−/S4+ downregulated genes*	Benjamini		EB downregulated genes	Benjamini
1	multicellular organismal development	6.00E-15	1	immune response	5.40E-09
2	developmental process	2.70E-14	2	response to wounding	9.20E-08
3	multicellular organismal process (9)	2.40E-13	3	response to external stimulus	2.70E-07
4	system development (18)	8.20E-12	4	defense response	3.40E-07
5	anatomical structure development (25)	1.10E-10	5	immune system process	4.80E-07
6	organ development (20)	2.50E-10	6	positive regulation of biological process	2.70E-06
7	response to external stimulus (3)	2.10E-09	7	inflammatory response	4.80E-06
8	positive regulation of biological process (6)	5.10E-09	8	response to stimulus	7.30E-06
9	organ morphogenesis	7.50E-09	9	multicellular organismal process	1.50E-05
10	cell-cell signaling	1.50E-08	10	positive regulation of cellular process	5.70E-05
11	regulation of cell proliferation (11)	1.50E-08	11	regulation of cell proliferation	5.50E-05
12	cell communication	5.50E-08	12	positive regulation of immune system process	6.10E-04
13	positive regulation of cellular process	5.40E-08	13	response to stress	8.40E-04
14	immune system process (1)	1.40E-07	14	regulation of response to stimulus	7.80E-04
15	anatomical structure morphogenesis	3.90E-07	15	regulation of immune system process	1.20E-03

* Number in the bracket represents the position of gene ontology ranking in EB cells.

**Figure 7:**
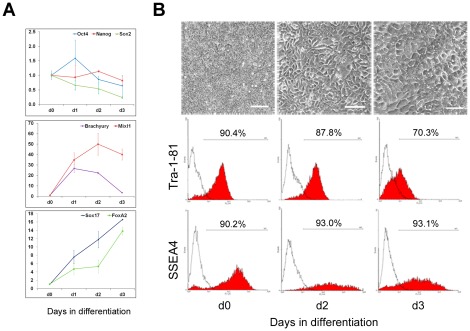
Differentiation of H1 hESCs to definitive endoderm. H1 hESCs were treated with high levels of Activin A and LY294002 for 3 days as detailed in Materials and Methods. The cells were stained with Tra-1-81 and SSEA4 antibodies and analysed for their gene expression by q-RT-PCR at the indicated time points during the differentiation. (A) Expression of marker genes were analysed by qRT-PCR at the indicated time points during the differentiation. (B) Phase-contrast images and histogram of flow cytometry analysis on Tra-1-81 and SSEA4 antibody staining at the indicated time points. Scale bar = 100 µm.

Since cells in the early neural differentiation and early EB formation share a similar gene expression pattern and phenotype, we asked whether early mesendoderm differentiation would also generate this population. To address this question, we differentiated H1 hESCs into definitive endoderm using two different methods (Activin A with sodium butyrate or LY294002, see Materials and Methods for details). Both differentiation procedures exhibited a similar phenotype and gene expression pattern, in which expression of Sox17 and FoxA2 steadily increased over the 3-day differentiation period, whereas, after an initial upregulation, expression of the mesendoderm markers, brachyury and Mixl1, were downregulated by day 3 (Figure 7A). Furthermore, Sox2 expression was continuously downregulated (Figure 7A). This gene expression pattern is consistent with our previous data and existing published data on endoderm differentiation [6], [7], [27], indicating that the majority of these cells have differentiated to mesendoderm by day 1–2 of the differentiation and are committed to endoderm by day 3. However, during the differentiation, both Tra-1-81 and SSEA4 were continuously expressed in a considerable proportion of cells, approximately 90% and 70% after 2 and 3 days of differentiation, respectively (Figure 7B). These results demonstrated that differentiated mesendoderm and early endoderm cells can also express TRA-1-81 and SSEA4, which is in line with a previous report that found Tra-1-60 and SSEA4 positive cells in a proportion of Sox17+ cells that could only be differentiated into endoderm and mesoderm [28]. As a whole, these results therefore indicate that differentiation towards the neural lineage may share a similar initial process to differentiation via EB formation that is distinct from that of high-dose Activin-induced definitive endoderm differentiation.

## Discussion

Differentiation of hESCs to specific cell lineages is a complex process involving multiple stages. An improved understanding of this elaborate process will provide further insights into the molecular control of early developmental processes, which is fundamental for the realization of targeted drug and cell therapies. In this study, we focused on the initial stage of hESC neural differentiation and have identified a transient cell population by carefully monitoring the expression of hESC stage-specific surface antigens. These cells no longer express the hESC specific marker Tra-1-81 but retain expression of SSEA4, another hESC marker.

Although TR−/S4+ cells are able to form EB-like structures and can differentiate into non-neural lineages (Figure 5), they are distinct from their original undifferentiated hESCs. They do not express Tra-1-81, a marker for undifferentiated hESCs [3], [17], and they exhibit an altered gene expression profile when compared to hESCs. Undifferentiated hESCs express high levels of pluripotent genes (*Oct4*, *Nanog* and *LRH-1*) but do not express neural lineage markers (Pax6, nestin and Sox1), whereas TR−/S4+ cells express both pluripotent and neural markers, although these are at low levels. Furthermore, their response to extrinsic signals also differs from hESC. When reintroduced into hESC self-renewal culture conditions, they were unable to maintain the low-level co-expression of both Oct4 and Pax6 with Tra-1-81(−)/SSEA4(+) identity, nor were they able to revert back to a Tra-1-81(+)/SSEA4(+) hESC state expressing high levels of Oct4. Instead, they differentiated into mixed neural and non-neural lineages. On the other hand, these cells are also different from committed NPCs. Fully differentiated neural progenitors do not express pluripotent genes, but do express high levels of neural markers, whereas the TR−/S4+ cells co-express both pluripotent and neural markers. Therefore, these cells represent an intermediate stage between pluripotent hESCs and differentiated NPCs.

The TR−/S4+ cells share several similarities with the mouse primitive NSCs that were identified in studies in the initial stage of neural ectoderm differentiation/formation in mESCs and embryos [4], [29]. Both cell types were identified in the early stage of neural development and express pluripotent and neural genes in addition to FGF5. They are capable of producing not only neural progenitors but also non-neural lineage cells, depending on their environmental cues. However, TR−/S4+ cells do not share all the features of mouse primitive NSCs, the main difference being their response to LIF stimulation. LIF has been reported to stimulate an increase in proliferation in mouse primitive NSCs [4], whereas we found that TR−/S4+ cells were unresponsive to LIF and express very low levels of the LIF receptor (Figure 2C). This discrepancy may reflect differences in the signaling pathways that regulate and maintain self-renewal and differentiation of human and mouse ESCs. Mouse ESCs require LIF and BMP to maintain their self-renewal [30] and FGF signaling promotes their neural differentiation [31]. However, human ESCs require bFGF and Activin for their self-renewal [3], [32], with the inhibition of BMP and Activin leading to their neural differentiation [16], [20]. Interestingly, the TR−/S4+ cells also share similar changes in gene expression pattern with the cells of early EBs. This finding may further support the similarities between TR−/S4 cells and primitive ectoderm as EB differentiation, to a certain extent, resembles the *in vivo* development of the embryo in which primitive ectoderm is one of the first lineages to form [25]. It is also noteworthy that similar TR−/S4+ cells were not detected during endoderm differentiation. Although the underlying implication of this observation remains to be elucidated, this suggests that the differentiation process towards endoderm and neural lineages are distinct from each other and may additionally affect the expression of cell surface antigens. This finding is also supported by a recent report that showed changes in glycosphoingolipid composition during endoderm differentiation that were entirely different from those in neural differentiation and EB formation [33].

Our data also shows that prior to the establishment of a fully committed neural lineage (demonstrated by high expression of neural genes and an absence of pluripotent markers), changes in culture conditions can alter the differentiation outcome. For example, if the culture conditions are maintained in favor of neural differentiation, expression of neural genes, including Sox2, are further increased, whilst expression of Oct4 and Nanog are reduced, and cells are committed to neural progenitors. Otherwise, under different culture conditions, cells can be differentiated to a non-neural fate.

One interesting observation is the dynamic changes in expression of the pluripotent genes *Oct4* and *Sox2*. Both have been intensively studied as central regulators for the maintenance of ESC pluripotency [8], [13], [34], [35], and as key factors required to reprogram somatic cells to the embryonic state [36], [37]. However, it is unclear how or if these pluripotent factors play a role in the transition from a pluripotent cell to a lineage–specific cell type. In this study we have demonstrated that the downregulation of Oct4 during neural differentiation of hESCs is progressive and is accompanied by the gradual upregulation of neural markers (Figure 3). The relationship between Oct4 and neural genes appear to be negatively correlated, and it appears that the neural lineage cannot be fully established prior to complete repression of Oct4. By contrast, Sox2 is consistently expressed during this process and is upregulated in the neural progenitors. These data are in line with the recent finding [38] that Oct4 and Sox2 are differentially expressed during neural and mesendoderm differentiation and suggests that pluripotent genes may play a role during early differentiation of the embryo proper. Oct4 has been shown to function in mouse trophectoderm differentiation by interacting with the trophectoderm transcription factor Cdx2, the resulting complex of which enhances Cdx2 expression and represses Oct4 expression [39]. Similarly, in mesoderm and endoderm differentiation, Oct4 interacts with the Sox17 transcription factor to repress Sox2 expression and enhance Sox17 expression [40]. However, in these non-neural lineages, Oct4 expression was maintained, and even slightly upregulated during the initial stages of the differentiation, which is not the case during neural differentiation. Therefore, Oct4 may not be directly involved in neural differentiation, but may rather function as a gate-keeping gene to govern pluripotency [41]. By contrast, Sox2 has been found to play a critical role in the maintenance of neural progenitor stem cells [42]. In keeping with this, we showed that Sox2 expression remains high throughout neural differentiation, thus suggesting that Sox2 may be a key player in the initiation of neural differentiation. However, how Sox2 regulates the neural initiation and, in particular, its relationship with Oct4 during this process remains to be elucidated. As such, these TR−/S4+ cells will be both a valuable and useful cell type for such an investigation.

## Materials and Methods

### Culture and differentiation of hESCs

Human embryonic stem cell lines H1 and H7 (WiCell) were routinely cultured on matrigel-coated plates using mouse embryonic fibroblast-conditioned medium (MEF-CM) supplemented with 8 ng/ml bFGF (PeproTech) and propagated mechanically in 1∶3 ratio after the treatment with collagenase IV [43]. Neural differentiation was carried out in N2B27 medium supplemented with 100 ng/ml noggin (R&D systems) alone [16] or with 500 ng/ml noggin and 10 µM SB431542 [20], [21] in poly-L-lysine/laminin or matrigel coated plates. After formation of neural progenitor cells, noggin was replaced by bFGF (20 ng/ml) and NPCs were maintained in this condition and propagated with TrypLE (Invitrogen). Neurons and glia were differentiated by withdrawal of bFGF. Differentiation via cell aggregate formation was carried out as described previously [44], with 1×10^4^ cells/aggregate in 96-well plate. Aggregates were transferred into a 24 well plate and cultured in suspension for a week. They were then mechanically dissociated and cultured in gelatin-coated plates/coverslips. Definitive endoderm differentiation was performed using two different protocols: 1) hESCs were treated with 100 ng/ml Activin A and 0.5–1 mM sodium butyrate as described previously [7]; 2) the hESCs were treated with 100 ng/ml Activin A (PeperoTech) and 20 µM LY294002 (Sigma) in RPMI1641-B27 medium for 1 day followed by 100 ng/ml Activin A and 10 µM LY294002 for another 2 days supplemented with 0.1% insulin-transferrin-selenium (ITS, Sigma).

### Immunocytochemistry and Western blotting

Cells were fixed at room temperature with 4% paraformaldehyde for 10 minutes. Non-specific proteins were blocked by incubation in PBS containing 10% goat serum (Sigma) and 0.1% Triton-X for one hour. The cells were then treated with primary antibodies overnight at 4°C. Following PBS washes, cells were incubated with fluorescence-conjugated secondary antibody for 30 minutes, and finally mounted to cover-slip with Mowiol. For cell surface antigens, cells were stained live (without fixation), incubated with primary antibody for 1 h at 4°C, washed with PBS and then incubated with the appropriate secondary antibody for 30 minutes at 4°C. The staining was visualized and captured with a Nikon Eclipse TC2000-U microscope or Leica TCS SP5.

For Western blotting, cells were lysed in pre-heated 1% sodium dodecyl sulphate (SDS) and homogenized by passing through a needle. The supernatant was collected after centrifugation and assayed for protein concentration using bicinchoninic acid protein assay kit (Pierce). 50 µg lysate from each sample were separated in 10% SDS-PAGE gel and transferred onto a polyvinylidene fluoride (PVDF) membrane. Membranes were blocked for 1 h in Tris-buffered saline (TBS) containing 0.1% Tween (TBST) and 5% milk prior to incubation with the appropriate primary antibody overnight at 4°C. Membranes were washed twice with TBST and incubated with HRP-conjugated secondary antibodies at a dilution of 1∶2000 for 1 h at room temperature and were visualized by enhanced chemiluminescence (Pierce). All antibodies are listed in Table S1.

### Flow cytometry analysis and cell sorting

Cells were detached into single cells by trypsin/EDTA, incubated with antibody directly (for surface antigens) or following fixation with 4% paraformaldehyde (for 15 min) and permeation with 100% ethanol (2 min) (for nuclear proteins). Cells were analysed using a BD FACSCalibur and CELLQUEST software. Fluorescence-activated cell sorting (FACS) was carried out with BD FACSAria II after staining live cells with antibodies against cell surface antigens. Magnetic-activated cell sorting was performed with Dynal magnetic beads (Invitrogen) following the manufacturer's instructions. Briefly, 25 µl (1×10 ^7^) Dynabeads were pre-coated with 1 µg primary antibody by incubation at 4°C for 30 minutes in 1 ml buffer 1. Pre-coated beads were then incubated with cells (0.5 ml) at 4°C for 30 minutes with gentle tilting and rotation. 2 ml buffer 1 was added into the tube to limit trapping of unbound cells before placing the tube onto the magnet for 2 minutes. For negative selection, the supernatant, containing the unbound cells, was transferred to a fresh tube for further experiments. For positive isolation, the bead-bound cells were gently washed with buffer 1 and collected in appropriate solution/medium for further experiments.

### Gene expression analysis by quantitative RT-PCR (qRT-PCR)

Total RNA was extracted using TRI reagent solution (Sigma) following the manufacturer's instructions. Remaining traces of DNA were removed by DNase I treatment (Invitrogen). Reverse transcription and qPCR were performed as described previously [45]. RNA without reverse transcription was used as a negative control. The relative gene expression levels were calculated by calibrating their Ct values with those of housekeeping genes, HPRT and GAPDH, and then normalized to undifferentiated hESCs. The standard deviation was calculated from at least four qPCRs from three independent experiments. Primer sequences are listed in Table S2.

### Global gene expression analysis

RNA-sequencing data was generated as previously reported [22]. Microarray data was obtained from publically available database [26]. Significantly upregulated or downregulated genes (≥5-folds) were selected and analysed using DAVID bioinformatics resources [46].

## Supporting Information

Figure S1 **Expression of Tra-1-81 and SSEA4 during neural differentiation of hESCs with dual SMAD inhibition protocol.** Neural differentiation with dual SMAD inhibition protocol also exhibits the sequential loss of Tra-1-81 and SSEA4. Flow cytometry histogram showing Tra-1-81 and SSEA4 staining in hESCs and at day 9 of the neural differentiation.
(TIF)

Table S1 **Antibodies used for immunocytochemistry, FACS and Western blotting.**
(DOCX)

Table S2 **Primer sequences for RT-PCR.**
(DOCX)
