# Supplementary figures and images for: Identification and Characterisation of the Early Differentiating Cells in Neural Differentiation of Human Embryonic Stem Cells
Source: PLoS One. 2012 May 15;7(5):e37129. doi: 10.1371/journal.pone.0037129 (PMC3352872; doi:10.1371/journal.pone.0037129)

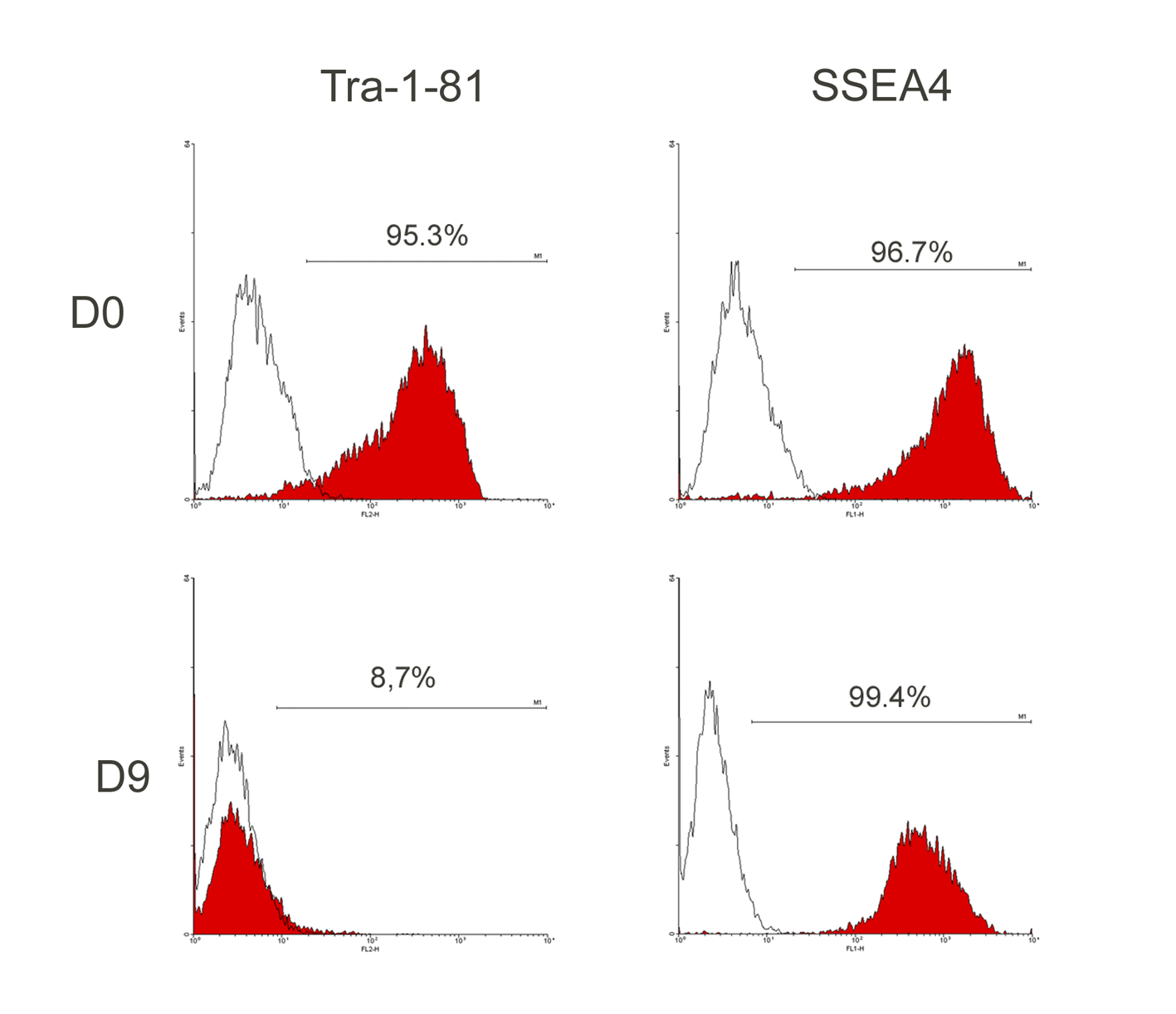

Supplement: Figure S1 — Expression of Tra-1-81 and SSEA4 during neural differentiation of hESCs with dual SMAD inhibition protocol. Neural differentiation with dual SMAD inhibition protocol also exhibits the sequential loss of Tra-1-81 and SSEA4. Flow cytometry histogram showing Tra-1-81 and SSEA4 staining in hESCs and at day 9 of the neural differentiation. (TIF) [file pone.0037129.s001.tif]
