# Supplementary material for: Identification and Characterisation of the Early Differentiating Cells in Neural Differentiation of Human Embryonic Stem Cells
Source: PLoS One. 2012 May 15;7(5):e37129. doi: 10.1371/journal.pone.0037129 (PMC3352872; doi:10.1371/journal.pone.0037129)
Supplement: Table S1 — Antibodies used for immunocytochemistry, FACS and Western blotting. (DOCX) [file pone.0037129.s002.docx]

**Table S1. Antibodies used for immunocytochemistry, FACS and Western blotting.**

| **Primary antibodies** | | |  |  |  |
| --- | --- | --- | --- | --- | --- |
| **Antigen Name** | **Type** | | **Company** | **Cat#** | **Dilution** |
| AFP | mouse monoclonal | | Sigma | A8452 | IF: 1:500 |
| b-actin | Rabbit polyclonal | | abcam | AB8227 | WB: 1:5000 |
| b-Tub III | mouse monoclonal | | Sigma | T8660 | IF: 1:1000 |
| GATA6 | Rabbit polyclonal | | Santa Cruz | sc-9055 | IF: 1:50 |
| GFAP | Rabbit polyclonal | | DAKO | Z0334 | IF: 1:500 |
| HNF4a | Rabbit polyclonal | | Santa Cruz | sc-8987 | IF: 1:200 |
| MAP2 | Rabbit polyclonal | | millipore | AB5622 | IF: 1:1000 |
| muscle actin | mouse monoclonal | | DAKO | M0635 | IF: 1:50 |
| Nanog | Rabbit polyclonal | | abcam | AB21624 | WB: 1:500 |
| Nestin | mouse monoclonal | | millipore | MAB5326 | IF: 1:200 |
| Oct4 | Rabbit polyclonal | | abcam | AB19857 | IF: 1:100; WB: 1:500 |
| Pax6 | Rabbit polyclonal | | millipore | AB5409 | IF: 1:1000 |
| SSEA1 | mouse monoclonal | | DSHB | MC480 | IF: 1:5 |
| SSEA4 | mouse monoclonal | | DSHB | MC813-70 | IF: 1:5 |
| Sox1 | Rabbit polyclonal | | abcam | AB22572 | IF: 1:200 |
| Tra-1-81 | mouse monoclonal | | Santa Cruz | sc-21706 | IF: 1:100 |
|  |  | |  |  |  |
| **Secondary antibodies** | | |  |  |  |
| goat anti-rabbit IgG-HRP | | | Santa Cruz | sc-2004 | WB: 1:2000 |
| Alexa flour goat anti-mouse IgG 488 | | | Invitrogen | A11029 | IF: 1:400 |
| Alexa flour goat anti-mouse IgG 568 | | | Invitrogen | A11004 | IF: 1:400 |
| Alexa flour goat anti-rabbit IgG 488 | | | Invitrogen | A21426 | IF: 1:400 |
| Alexa flour goat anti-mouse IgG 555 | | | Invitrogen | A21426 | IF: 1:400 |
| goat anti-mouse IgG-FITC | | | Santa Cruz | sc-2081 | IF: 1:100 |
| goat anti-mouse IgM-PE | | | Santa Cruz | sc-3768 | IF: 1:100 |
|  | |  |  |  |  |
| **Isotype controls** | |  |  |  |  |
| normal mouse IgG | |  | Santa Cruz | sc-3880 | 1:10(IF) |
| normal mouse IgM | | | Santa Cruz | sc-3881 | 1:10(IF) |
