# Supplementary material for: Identification and Characterisation of the Early Differentiating Cells in Neural Differentiation of Human Embryonic Stem Cells
Source: PLoS One. 2012 May 15;7(5):e37129. doi: 10.1371/journal.pone.0037129 (PMC3352872; doi:10.1371/journal.pone.0037129)
Supplement: Table S2 — Primer sequences for RT-PCR. (DOCX) [file pone.0037129.s003.docx]

**Table S2. Primer sequences for RT-PCR**

| **Gene** | **Forward primer (5’-3’)** | **Reverse primers (5’-3’)** |
| --- | --- | --- |
| AFP | TGGGACCCGAACTTTCCA | GGCCACATCCAGGACTAGTTTC |
| ALB | GCACAGAATCCTTGGTGAACAG | ATGGAAGGTGAATGTTTTCAGCA |
| Brachyury | TGCTTCCCTGAGACCCAGTT | GATCACTTCTTTCCTTTGCATCAAG |
| FGF5 | CAGCACCAAAGGCTCAGCTT | CCTTGCTTCTAACCCATCATATCC |
| GAPDH | TCTGCTCCTCCTGTTCGACA | AAAAGCAGCCCTGGTGACC |
| GATA6 | ACTTGAGCTCGCTGTTCTCG | CAGCAAAAATACTTCCCCCA |
| GSC | GAGGAGAAAGTGGAGGTCTGGTT | CTCTGATGAGGACCGCTTCTG |
| HPRT | TCCTTGGTCAGGCAGTATAATCC | GTCAAGGGCATATCCTACAACAAA |
| LIFR | CTGGAACAGGCCGTGGTAC | ACTCCACTCTTCGAGACCAG |
| LRH-1 | CGAGTGGGCCAGGAGTAGTA | CGGTAAATGTGGTCGAGGAT |
| MEOX1 | CCAGGACGAACTCCTCGTCAGC | CAGTGCTCCCAAGCACCCCG |
| Nanog | TGATTTGTGGGCCTGAAGAAAA | GAGGCATCTCAGCAGAAGACA |
| Nestin | GAGGGAAGTCTTGGAGCCAC | AAGATGTCCCTCAGCCTGG |
| Pou5F1 (Oct4) | TCGAGAACCGAGTGAGAGGC | CACACTCGGACCACATCCTTC |
| Pax6 | TCCGTTGGAACTGATGGAGT | GTTGGTATCCGGGGACTTC |
| Rex1 | GGTGGCATTGGAAATAGCAG | TGCCTAGTGTGCTGGTGGT |
| Sox1 | AACACTTGAAGCCCAGATGGA | GCAGGCTGAATTCGGTTCTC |
| Sox2 | GCCGAGTGGAAACTTTTGTCG | GCAGCGTGTACTTATCCTTCTT |
